# Polymyxin B/Tigecycline Combination vs. Polymyxin B or Tigecycline Alone for the Treatment of Hospital-Acquired Pneumonia Caused by Carbapenem-Resistant *Enterobacteriaceae* or Carbapenem-Resistant *Acinetobacter baumannii*

**DOI:** 10.3389/fmed.2022.772372

**Published:** 2022-06-10

**Authors:** Kang Chang, Haibo Wang, Jianping Zhao, Xianghong Yang, Bo Wu, Wenkui Sun, Man Huang, Zhenshun Cheng, Hong Chen, Yuanlin Song, Ping Chen, Xiangqi Chen, Xin Gan, Wanli Ma, Lihua Xing, Yimin Wang, Xiaoying Gu, Xiaohui Zou, Bin Cao

**Affiliations:** ^1^National Clinical Research Center for Respiratory Diseases, Clinical Center for Pulmonary Infections, China-Japan Friendship Hospital, Capital Medical University, Beijing, China; ^2^Peking University Clinical Research Institute, Peking University First Hospital, Beijing, China; ^3^Department of Respiratory and Critical Care Medicine, Tongji Medical College, Tongji Hospital, Huazhong University of Science and Technology, Wuhan, China; ^4^Department of Intensive Care Unit, Zhejiang Provincial People’s Hospital, People’s Hospital of Hangzhou Medical College, Hangzhou, China; ^5^Department of Respiratory and Critical Care Medicine, Wuxi People’s Hospital Affiliated to Nanjing Medical University, Wuxi, China; ^6^Department of Respirology and Critical Care Medicine, The First Affiliated Hospital of Nanjing Medical University, Nanjing, China; ^7^Department of General Intensive Care Unit, The Second Affiliated Hospital of Zhejiang University School of Medicine, Hangzhou, China; ^8^Department of Pulmonary and Critical Care Medicine, Zhongnan Hospital of Wuhan University, Wuhan, China; ^9^Department of Respiratory and Critical Care Medicine, Ruijin Hospital, Shanghai Jiao Tong University School of Medicine, Shanghai, China; ^10^Department of Pulmonary and Critical Care Medicine, Zhongshan Hospital, Fudan University, Shanghai, China; ^11^Department of Respiratory Medicine, The Second Xiangya Hospital, Central South University, Changsha, China; ^12^Department of Respiratory Medicine, Fujian Medical University Union Hospital, Fujian Medical University, Fuzhou, China; ^13^Department of Respiratory and Critical Care Medicine, The First Affiliated Hospital of Nanchang University, Nanchang, China; ^14^Department of Respiratory and Critical Care Medicine, Tongji Medical College, Union Hospital, Huazhong University of Science and Technology, Wuhan, China; ^15^Department of Pulmonary and Critical Care Medicine, The First Affiliated Hospital of Zhengzhou University, Zhengzhou, China; ^16^Department of Pulmonary and Critical Care Medicine, China Centre of Respiratory Medicine, National Clinical Research Centre for Respiratory Diseases, China-Japan Friendship Hospital, Beijing, China; ^17^Institute of Respiratory Medicine, Chinese Academy of Medical Sciences, Peking Union Medical College, Beijing, China; ^18^School of Medicine, Tsinghua University, Beijing, China

**Keywords:** polymyxin B, tigecycline, carbapenem-resistant organism, carbapenem-resistant *Enterobacteriaceae*, hospital-acquired pneumonia

## Abstract

**Introduction:**

It is not clear whether polymyxin B/tigecycline (PMB/TGC) combination is better than PMB or TGC alone in the treatment of hospital-acquired pneumonia (HAP) caused by carbapenem-resistant organisms (CROs).

**Methods:**

We conducted a multicenter, retrospective cohort study in patients with HAP caused by CROs. The primary outcome was 28-day mortality, and the secondary outcomes included clinical success and the incidence of acute kidney injury (AKI). Multivariate Cox regression analysis was performed to examine the relationship between antimicrobial treatments and 28-day mortality by adjusting other potential confounding factors.

**Results:**

A total of 364 eligible patients were included in the final analysis, i.e., 99 in the PMB group, 173 in the TGC group, and 92 in the PMB/TGC combination group. The 28-day mortality rate was 28.3% (28/99) in the PMB group, 39.3% (68/173) in the TGC group, and 48.9% (45/92) in the PMB/TGC combination group (*p* = 0.014). The multivariate Cox regression model showed that there was a statistically significant lower risk of 28-day mortality among participants in the PMB group when compared with the PMB/TGC combination group [hazard ratio (HR) 0.50, 95% confidence interval (CI) 0.31–0.81, *p* = 0.004] and that participants in the TGC group had a lower risk of 28-day mortality than in the PMB/TGC combination group but without statistical significance. The incidence of AKI in the PMB group (52.5%) and the PMB/TGC combination group (53.3%) was significantly higher than that in the TGC group (33.5%, *p* = 0.001).

**Conclusion:**

The appropriate PMB/TGC combination was not superior to appropriate PMB therapy in the treatment of HAP caused by carbapenem-resistant *Enterobacteriaceae*/carbapenem-resistant *Acinetobacter baumannii* (CRE/CRAB) in terms of 28-day mortality.

## Introduction

Antimicrobial resistance is one of the most serious public health challenges in the world, especially carbapenem resistance. Carbapenem-resistant organisms (CROs), such as carbapenem-resistant *Acinetobacter baumannii* (CRAB) and *Enterobacteriaceae* (CRE), are among the “urgent threats” according to the Centers for Disease Control and Prevention (CDC), which should be addressed appropriately (1–3). The mortality of invasive CRE infections usually exceeds 30% (4). The treatment options are very limited for CROs because they are often resistant not only to carbapenems but also to most other antibiotics available (5, 6).

One of the strategies for managing CRO infections is to use the existing antibiotics wisely and rationally to improve clinical efficacy and meanwhile reduce adverse events. For example, polymyxins are often used in combination with tigecycline (TGC) or carbapenems to treat CRO infections (7–10), even though randomized controlled trials (RCTs) have confirmed that colistin/meropenem or colistin/rifampicin combinations are not superior to colistin alone in the treatment of carbapenem-resistant Gram-negative bacilli or drug-resistant *A. baumannii* (11, 12). However, only a few clinical studies have been conducted on polymyxin B/TGC (PMB/TGC) combination in treating CRO infections (13, 14). The optional choice against hospital-acquired pneumonia (HAP) caused by CRO is still controversial and whether or not PMB/TGC should be used as monotherapy or combination therapy is also unclear (15).

In this study, we retrospectively identified the patients with HAP caused by CRO who were initially treated with appropriate PMB and/or TGC. We aimed to evaluate the efficacy and safety of appropriate PMB/TGC combination therapy in treating HAP caused by CRO when compared with appropriate PMB or TGC alone.

## Materials and Methods

### Study Design and Setting

This is a multicenter, retrospective cohort study. The patients were enrolled from 14 tertiary general hospitals across China (see Supplementary Table 4). The study protocol was approved by the Ethics Committee of China-Japan Friendship Hospital (no. 2018-146-K103), which was endorsed by a rapid ethic review in each participating center. Researchers waived the need to obtain written informed consent due to its retrospective characteristic.

The patients with HAP due to CRO who were treated with PMB and/or TGC during the period from 1 January 2018 to 31 May 2020 were identified. Relevant data were retrieved from the electronic medical record system. The patients were followed up to document the survival status and safety data until at least day 28 after the onset of infection (day 1).

### Study Population

The patients were included in this study only when they were diagnosed with HAP due to CRO and received appropriate PMB and/or TGC therapy (for PMB, intravenous and/or nebulized administration were appropriate). It is reported that the early use of antibiotics in septic patients can reduce the in-hospital mortality (16). This phenomenon suggests that even for the same two antibiotics, the different intervals between the two drugs may lead to different clinical outcomes, so we can justifiably consider them as two different treatment strategies. Therefore, in this study, we included the interval between antibiotic used as one of the criteria to define appropriate therapy. PMB or TGC therapy was considered appropriate when the treatment lasted for ≥5 days in the patients surviving more than 5 days or ≥48 h in the patients surviving ≤5 days (12). Moreover, PMB and/or TGC were administered at recommended dose ranges within 5 days after the onset of infection or within 3 days after knowing the susceptibility testing results; or PMB and/or TGC were used empirically at the recommended doses (17, 18) until 3 days after knowing the susceptibility testing results. We also believe that it was reasonable if clinicians refer to drug instructions for administration. The patients were excluded if they received inappropriate PMB and inappropriate TGC therapies, key missing data prevented study evaluation, or patients died within 48 h after infection. The patients were assigned to PMB group when only PMB was used appropriately, TGC group when only TGC was used appropriately, or PMB/TGC group when both PMB and TGC were used appropriately.

### Data Collected

The data downloaded from the electronic medical record system were de-identified and used for further analyses. The database was reviewed to ensure that the inclusion and exclusion criteria were met. The primary outcome was 28-day all-cause mortality since the onset of infection (day 1). The patients or their family members were interviewed by telephone if day 28 follow-up data were missing in the information system. The secondary outcome was the clinical success rate. We also analyzed the incidence of acute kidney injury (AKI) as an adverse event about PMB used. All potential confounding variables were collected, such as demographics, underlying conditions, Charlson Comorbidity Index (CCI) (19), mechanical ventilation, laboratory tests, acute physiology, and chronic health evaluation II (APACHE II) score (20), sequential organ failure assessment (SOFA) score on day 1 (21), microbiological data, antimicrobial treatment, and AKI. The data were clarified *via* rechecking the source document if there was any discrepancy. The clinical isolates were identified and tested for susceptibility at each participating center. The susceptibility testing results were interpreted according to the 2020 Clinical and Laboratory Standards Institute (CLSI) (22) or Food and Drug Administration (FDA) breakpoints (23) to confirm CRO pathogens. The antimicrobial agent was considered active if the pathogen was “susceptible” or “intermediate” in susceptibility testing.

### Definitions

Hospital-acquired pneumonia was defined as pneumonia not incubating at the time of hospital admission and occurring at 48 h or more after admission. The clinical diagnosis of pneumonia was based on new or progressive infiltrates, consolidations, ground-glass opacities on chest X-ray or CT, and 2 or more of the following symptoms: (1) fever, body temperature >38°C; (2) purulent airway secretions; and (3) peripheral blood white blood cell count >10 × 10^9^/L or <4 × 10^9^/L (24). In addition, for the purpose to minimize the possibility of colonization, target pathogens must be those isolated from qualified lower respiratory tract secretions [the number of neutrophils >25 per low-power field (LPF), the number of epithelial cells <10/LPF, or the ratio between neutrophils and epithelial cells >2.5:1], protected specimen brush (PSB), bronchoalveolar lavage fluid (BALF), and lung tissue or sterile body fluids and were consistent with clinical manifestations (25). Clinical success was defined as a composite of patient survival, hemodynamic stability (systolic blood pressure >90 mmHg without vasopressor support), SOFA score improved by at least 30% if baseline SOFA ≥3 or stable if baseline SOFA <3, and oxygenation index stable or improved (12). CRE is defined as a susceptibility test showing resistance to at least one of ertapenem, doripenem, meropenem, or imipenem in the *Enterobacteriaceae* family. CRAB or carbapenem-resistant *Pseudomonas aeruginosa* (CRPA) refers to *A. baumannii* or *P. aeruginosa* that is intermediate or resistant to at least one of doripenem, meropenem, or imipenem by susceptibility results and if minimum inhibitory concentration (MIC) values were not available, the report of the local laboratory was adopted (26, 27). AKI, sepsis, and septic shock were defined as previously reported (28–30).

### Statistical Analysis

SAS software (version 9.4, SAS Institute, Cary, NC, United States) was used for statistical analysis. The baseline characteristics were compared among three groups using a Chi-square or Fisher’s exact test for categorical variables, and analysis of variance (ANOVA) or Kruskal–Wallis *H* test for continuous variables. The 28-day mortality rate was compared between treatment groups using the Cox proportional hazard model in terms of hazard ratio (HR) and 95% confidence interval (CI). Data were censored at the last contact date for patients who were lost to follow-up. Stratified analysis was performed by sex, comorbidity, vasopressor use, sepsis, mechanical ventilation, and pathogen. Subgroup analyses were based on important demographic characteristics and confounders, such as comorbidities, sepsis, vasoactive drugs, and pathogens, and were decided *a priori*. A multivariate Cox proportional hazard model was built by adjusting for the known risk factors associated with 28-day mortality or the significant variables identified in univariate analysis. The proportional hazards assumption was tested using Schoenfeld residuals, with *p* < 0.05 indicating non-proportionality. Kaplan–Meier method was used to estimate the curves of 28-day mortality in each treatment group. All *p*-values were two-sided and *p* < 0.05 was considered statistically significant.

## Results

### Patients and Treatment Details

**FIGURE 1:**
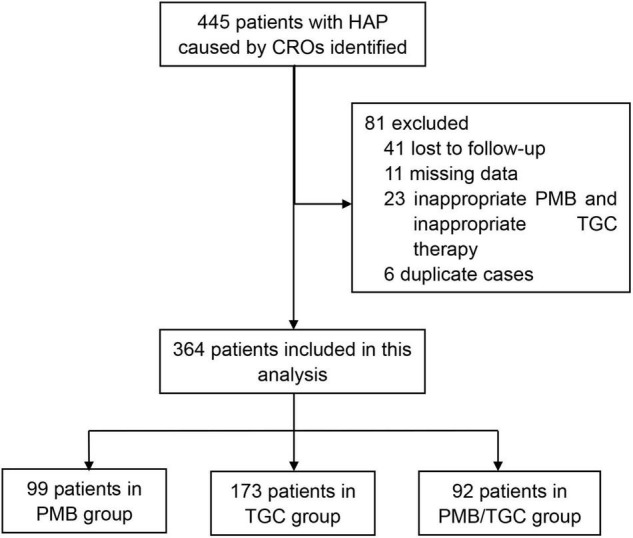
Flow chart for patient enrollment. HAP, hospital-acquired pneumonia; CRO, carbapenem-resistant organisms; PMB, polymyxin B; TGC, tigecycline.

A total of 445 patients with HAP caused by CRO were initially retrieved from the participating hospitals. Some of the patients were excluded due to inappropriate PMB and inappropriate TGC therapies (*n* = 23), missing data (*n* = 11), duplicate cases (*n* = 6), and unknown survival status (*n* = 41). Finally, 364 patients were eligible for this study, i.e., 99 in the PMB group, 173 in the TGC group, and 92 in the PMB/TGC combination group (Figure 1). About 1% (1/99) of the patients in the PMB group received PMB by nebulization and 1% (1/99) by intravenous plus nebulized administration; 1.3% (1/78) of the patients in the TGC group received PMB by nebulization and 2.6% (2/78) of patients received PMB by intravenous plus nebulized administration; and 2% (2/92) of the patients in combination therapy group received PMB by nebulization and 1% (1/92) of the patients received PMB by intravenous plus nebulized administration. There was no significant difference between the treatment groups (*p* = 0.859, data not shown).

Patients were predominantly men (75.3%). Significantly more patients had underlying respiratory diseases in the PMB group (59.6%) than in the TGC group (40.5%) or the PMB/TGC combination group (44.6%; *p* = 0.009). The baseline SOFA score was significantly higher in the PMB/TGC combination group (*p* = 0.004). Significantly more patients in the PMB/TGC combination group had septic shock (*p* = 0.005) and used vasoactive drugs (*p* = 0.007). Other clinical parameters were comparable between the three treatment groups (Table 1).

**TABLE 1:** Baseline characteristics of patients in terms of polymyxin B and/or tigecycline treatment.

Characteristic	Polymyxin B (*N* = 99)	Tigecycline (*N* = 173)	Polymyxin B/tigecycline (*N* = 92)	*p*-value
**Demographics**				
Sex, male	74 (74.7)	126 (72.8)	74 (80.4)	0.390^¶^
Age, years	63.00 ± 18.00	64.00 ± 18.00	58.00 ± 20.50	0.005^‡^
Body mass index	22.04 ± 4.87	22.89 ± 4.60	22.09 ± 4.56	0.346^‡^
Charlson Comorbidity Index	2.00 ± 2.00	1.00 ± 2.00	2.00 ± 2.00	0.739^‡^
**Underlying diseases**				
Chronic respiratory disease	59 (59.6)	70 (40.5)	41 (44.6)	0.009^¶^
Chronic cardiovascular disease	49 (49.5)	103 (59.5)	42 (45.7)	0.066^¶^
Chronic kidney disease	17 (17.2)	23 (13.3)	9 (9.8)	0.326^¶^
**Severity of illness at diagnosis of HAP due to CROs**				
SOFA score	5.00 ± 5.00	6.00 ± 5.00	7.00 ± 5.00	0.004^‡^
APACHE II score	17.00 ± 11.00	16.00 ± 10.00	18.00 ± 10.00	0.160^‡^
Vasopressor use	23 (23.2)	43 (24.9)	38 (41.3)	0.007^¶^
White blood cell count (×10^12^/L)	11.70 ± 7.91	10.16 ± 7.25	11.32 ± 8.27	0.360^‡^
Platelet count (×10^9^/L)	182.00 ± 173.00	136.00 ± 119.00	108.00 ± 149.00	0.002^‡^
Platelet count <15 ×10^9^/L	3 (3.0)	3 (1.7)	5 (5.4)	0.246^ξ^
Creatinine (μmol/L)	69.20 ± 75.35	65.70 ± 58.20	75.10 ± 58.70	0.310^‡^
Bilirubin (μmol/L)	13.82 ± 15.50	15.30 ± 14.24	15.38 ± 19.37	0.978^‡^
PaO_2_/FiO_2_ ratio	224.18 ± 126.33	204.44 ± 147.23	170.40 ± 157.94	0.002^‡^
Sepsis	41 (41.4)	71 (41.0)	35 (38.0)	0.005^¶^
Septic shock	18 (18.2)	38 (22.0)	36 (39.1)	
**Clinical details**				
Non-invasive mechanical ventilation	49 (49.5)	71 (41)	43 (46.7)	0.366^¶^
Invasive mechanical ventilation	83 (83.8)	130 (75.1)	78 (84.8)	0.092^¶^
Duration of non-invasive mechanical ventilation (day)	12.00 ± 18.00	7.00 ± 14.00	14.00 ± 24.00	0.196^‡^
Duration of invasive mechanical ventilation (day)	18.00 ± 24.00	15.00 ± 22.00	18.50 ± 19.00	0.279^‡^
Extracorporeal membrane oxygenation	17 (17.2)	22 (12.7)	22 (23.9)	0.067^¶^
Length of hospital stay (day)	26.00 ± 27.00	20.00 ± 24.00	19.00 ± 21.50	0.030^‡^
Length of stay before infection (day)	8.00 ± 17.00	6.00 ± 14.00	5.00 ± 14.00	0.618^‡^
Peak value of serum creatinine after treatment (μmol/L)	149.30 ± 154.10	104.20 ± 169.00	150.00 ± 155.20	0.016^‡^

*CRO, carbapenem-resistant organism; HAP, hospital-acquired pneumonia; SOFA, sequential organ failure assessment; APACHE, acute physiology and chronic health evaluation; PaO_2_, arterial oxygen partial pressure; FiO_2_, fractional inspired oxygen.*

*^¶^Chi-square test.*

*^‡^Kruskal–Wallis H test.*

*^ξ^Fisher’s exact test. Data are presented as median ± interquartile range or N (%).*

The most frequently isolated pathogens were CRAB (211/364, 58.0%), followed by carbapenem-resistant *Klebsiella pneumoniae* (CRKP) (142/364, 39.0%), CRPA (33/364, 9.0%), other CRE (6/364, 1.6%), and carbapenem-resistant *Escherichia coli* (6/364, 1.6%; Table 2). Due to missing susceptibility testing results in some of the study hospitals, we did not collect this information in its entirety. However, we analyzed the existing susceptibility results and found that both PMB and TGC maintained a high level of antibacterial activity against the target strains (see Supplementary Table 3).

**TABLE 2:** Antimicrobial therapies, pathogens, and prognosis of patients in terms of polymyxin B and/or tigecycline treatment.

Parameter	Polymyxin B (*N* = 99)	Tigecycline (*N* = 173)	Polymyxin B/tigecycline (*N* = 92)	*p*-value
**Antibiotics use**				
Polymyxin B*^a^*				
Time to treatment (day)*^b^*	3.00 ± 3.00	1.00 ± 9.00	3.00 ± 4.00	0.269^‡^
Duration of treatment (day)	14.00 ± 9.00	7.00 ± 8.00	12.00 ± 10.50	<0.0001^‡^
Drug used	99 (100%)	78 (45.1)	92 (100.0)	
**Tigecycline**				
Time to treatment (day)*^a^*	1.00 ± 6.00	1.00 ± 3.00	1.50 ± 2.00	0.315^‡^
Duration of treatment (day)	9.00 ± 15.00	13.00 ± 10.00	13.00 ± 10.00	0.005^‡^
Drug used	43 (43.4)	173 (100.0)	92 (100.0)	
**Pathogens at diagnosis of HAP due to CROs**				
Carbapenem-resistant *K. pneumoniae*	43 (43.4)	60 (34.7)	39 (42.4)	0.270^¶^
Carbapenem-resistant *E. coli*	2 (2.0)	1 (0.6)	3 (3.3)	0.249^ξ^
Other CRE	3 (3.0)	2 (1.2)	1 (1.1)	0.449^¶^
Carbapenem-resistant *A. baumannii*	49 (49.5)	114 (65.9)	48 (52.2)	0.013^¶^
Carbapenems-resistant *P. aeruginosa*	18 (18.2)	5 (2.9)	10 (10.9)	0.0001^¶^
Number of pathogens ≥2	17 (17.2)	12 (6.9)	13 (14.1)	0.026^¶^
**Outcomes**				
AKI before infection	25 (25.3)	32 (18.5)	25 (27.2)	0.205^¶^
AKI after infection	52 (52.5)	58 (33.5)	49 (53.3)	0.001^¶^
AKI Stage 1	18 (34.6)	24 (41.4)	17 (34.7)	0.514^¶^
AKI Stage 2	13 (25.0)	18 (31.0)	11 (22.4)	
AKI Stage 3	21 (40.4)	16 (27.6)	21 (42.9)	
Continuous renal replacement therapy	25 (25.3)	27 (15.6)	34 (37.0)	0.0005^¶^
Clinical success	41 (41.4)	59 (34.3)	25 (27.5)	0.130^¶^
28-day mortality	28 (28.3)	68 (39.3)	45 (48.9)	0.014^¶^
14-day mortality	18 (18.2)	46 (26.6)	28 (30.4)	0.129^¶^

*CRO, carbapenem-resistant organism; HAP, hospital-acquired pneumonia; CRE, carbapenem-resistant Enterobacteriaceae; AKI, acute kidney injury.*

*Data are presented as median ± interquartile range or N (%).*

*^a^Majority of patients received intravenous polymyxin B and there were no difference in nebulized polymyxin B used between the three groups.*

*^b^From day 1 to the start date of polymyxin B and/or tigecycline treatment.*

*^¶^Chi-square test.*

*^‡^Kruskal–Wallis H test.*

*^ξ^Fisher’s exact test.*

### Patient Outcome

**FIGURE 2:**
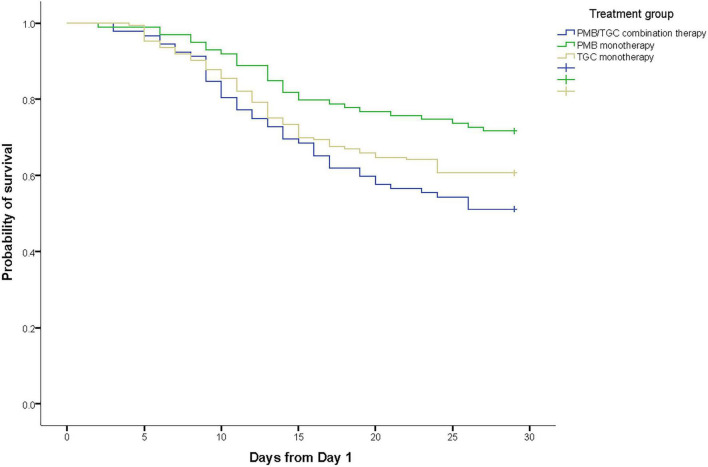
Kaplan–Meier survival estimates of patients with hospital-acquired pneumonia due to carbapenem-resistant organisms in terms of polymyxin B and/or tigecycline treatment.

Overall, 92 (25.3%) patients died within 14 days and 141 (38.7%) patients died within 28 days after the onset of infection (day 1). The 28-day mortality rate was 28.3% (28/99) in the PMB group, 39.3% (68/173) in the TGC group, and 48.9% (45/92) in the PMB/TGC combination group (*p* = 0.014; Table 2). Appropriate PMB therapy was associated with a significant survival advantage (HR 0.50, 95% CI 0.31–0.81, *p* = 0.004) when compared with PMB/TGC combination therapy, but not in the TGC group (HR 0.77, CI 95% 0.53–1.12, *p* = 0.169; Supplementary Table 2 and Figure 2). Appropriate PMB therapy resulted in the highest clinical success rate (41.4%) when compared with TGC (34.3%) and PMB/TGC (27.5%; Table 2).

Univariate analysis showed that chronic cardiovascular diseases (HR 1.69; 95% CI 1.2–2.39; *p* = 0.003), higher SOFA score on day 1 (HR 1.12; 95% CI 1.07–1.16; *p* < 0.001), higher APACHE II score on day 1 (HR 1.07; 95% CI 1.05–1.10; *p* < 0.001), sepsis (HR 1.51; 95% CI 0.99–2.30; *p* = 0.054), septic shock (HR 2.48; 95% CI 1.60–3.83; *p* < 0.001), pre-existing AKI before infection (HR 1.52; 95% CI 1.05–2.19; *p* = 0.027), and incident AKI after infection (HR 1.81; 95% CI 1.30–2.52; *p* = 0.0005) were risk factors for 28-day mortality (Supplementary Table 2). Clinical treatment success (HR 0.04; 95% CI 0.01–0.11; *p* < 0.0001), higher platelet count (HR 0.998; 95% CI 0.996–0.999; *p* = 0.010), and higher PaO_2_/FiO_2_ ratio (HR 0.998; 95% CI 0.996–0.999; *p* = 0.009) were associated with significant lower risk of 28-day mortality (Supplementary Table 2). The 28-day mortality was independent of pathogenic bacterial species and mixed infections caused by multiple pathogens (Supplementary Table 2). Subgroup analysis showed a survival benefit for appropriate PMB therapy over PMB/TGC combination therapy in most of the subgroups analyzed, except the patients with sepsis (or septic shock) or using vasoactive drugs, and the patients with CRBA infection (Table 3).

**TABLE 3:** Subgroup analyses.

Subgroup	No. of patients	Compared with polymyxin B/tigecycline	HR (95% CI)	*p*-Value
**Sex**				
Male	274	Polymyxin B	0.50 (0.30–0.85)	0.010
		Tigecycline	0.75 (0.50–1.15)	0.187
Female	90	Polymyxin B	0.55 (0.19–1.65)	0.288
		Tigecycline	0.89 (0.37–2.14)	0.793
**Underlying diseases**				
Chronic respiratory disease	170	Polymyxin B	0.47 (0.24–0.91)	0.025
		Tigecycline	0.98 (0.56–1.71)	0.930
Chronic cardiovascular disease	194	Polymyxin B	0.46 (0.25–0.84)	0.012
		Tigecycline	0.66 (0.41–1.06)	0.085
Chronic kidney disease	49	Polymyxin B	0.53 (0.16–1.74)	0.294
		Tigecycline	0.71 (0.24–2.07)	0.529
Acute kidney injury	82	Polymyxin B	0.50 (0.21–1.20)	0.122
		Tigecycline	1.01 (0.50–2.05)	0.983
**Vasopressor use (day 1)**				
Yes	104	Polymyxin B	0.92 (0.42–1.98)	0.824
		Tigecycline	1.23 (0.66–2.28)	0.514
No	260	Polymyxin B	0.39 (0.22–0.72)	0.002
		Tigecycline	0.64 (0.39–1.02)	0.063
**Sepsis and septic shock**				
Yes	239	Polymyxin B	0.63 (0.36–1.11)	0.109
		Tigecycline	1.05 (0.68–1.61)	0.836
No	125	Polymyxin B	0.33 (0.14–0.80)	0.014
		Tigecycline	0.37 (0.17–0.80)	0.012
**Invasive mechanical ventilation**				
Yes	291	Polymyxin B	0.51 (0.31–0.84)	0.008
		Tigecycline	0.87 (0.59–1.30)	0.496
No	73	Polymyxin B	0.39 (0.07–2.14)	0.280
		Tigecycline	0.54 (0.16–1.83)	0.321
**Pathogen**				
Carbapenem-resistant *K. pneumoniae*	217	Polymyxin B	0.47 (0.26–0.86)	0.014
		Tigecycline	0.70 (0.42–1.17)	0.171
Carbapenem-resistant *A. baumannii*	247	Polymyxin B	0.61 (0.34–1.09)	0.093
		Tigecycline	0.91 (0.57–1.44)	0.678

*HR, hazard ratio; CI, confidence interval.*

*All p-values were calculated by Cox proportional hazards regression model.*

The incidence of AKI was significantly higher in the PMB group (52.5%) and the PMB/TGC group (53.3%) than in the TGC group (33.5%, *p* = 0.001; Table 2). PMB-based therapies were associated with a relatively higher proportion of AKI Stage 3 or continuous renal replacement therapy (Table 2).

We built six different models to adjust the possible effects of confounding factors on mortality. The multivariate Cox proportional hazard model demonstrated that appropriate PMB therapy was associated with a significantly lower risk of 28-day mortality when compared with PMB/TGC combination therapy after adjustment for multiple established risk factors, such as age, sex, chronic respiratory disease, chronic cardiovascular disease, sepsis, SOFA score, APACHE II score, vasopressor use, platelet count, and creatinine. Multivariate analysis demonstrated that appropriate PMB therapy had a significant protective effect on mortality (Table 4).

**TABLE 4:** Model selection for multivariable analysis of 28-day mortality.

Model	Factors	HR (95% CI)	*p*-value
Crude	Polymyxin B vs. polymyxin B/tigecycline	0.50 (0.31–0.81)	0.004
	Tigecycline vs. polymyxin B/tigecycline	0.77 (0.53–1.12)	0.169
Model 1	Polymyxin B vs. polymyxin B/tigecycline	0.49 (0.30–0.79)	0.003
	Tigecycline vs. polymyxin B/tigecycline	0.74 (0.50–1.09)	0.126
Model 2	Polymyxin B vs. polymyxin B/tigecycline	0.48 (0.30–0.78)	0.003
	Tigecycline vs. polymyxin B/tigecycline	0.72 (0.49–1.05)	0.090
Model 3	Polymyxin B vs. polymyxin B/tigecycline	0.58 (0.36–0.94)	0.027
	Tigecycline vs. polymyxin B/tigecycline	0.82 (0.56–1.22)	0.328
Model 4	Polymyxin B vs. polymyxin B/tigecycline	0.55 (0.34–0.90)	0.017
	Tigecycline vs. polymyxin B/tigecycline	0.87 (0.59–1.29)	0.494
Model 5	Polymyxin B vs. polymyxin B/tigecycline	0.55 (0.34–0.90)	0.018
	Tigecycline vs. polymyxin B/tigecycline	0.88 (0.60–1.29)	0.507
Model 6	Polymyxin B vs. polymyxin B/tigecycline	0.58 (0.35–0.96)	0.035
	Tigecycline vs. polymyxin B/tigecycline	0.88 (0.59–1.31)	0.520

*HR, hazard ratio; CI, confidence interval.*

*Model 1: adjusted for age and sex; model 2: adjusted for age, sex, chronic respiratory disease, and chronic cardiovascular disease; model 3: adjusted for age, sex, chronic respiratory disease, chronic cardiovascular disease, and sepsis; model 4: adjusted for age, sex, chronic respiratory disease, chronic cardiovascular disease, sepsis, SOFA score, and APACHE II score; model 5: adjusted for age, sex, chronic respiratory disease, chronic cardiovascular disease, sepsis, SOFA score, APACHE II score, and vasopressor use; and model 6: adjusted for age, sex, chronic respiratory disease, chronic cardiovascular disease, sepsis, SOFA score, APACHE II score, vasopressor use, platelet count, and creatinine.*

*All p-values were calculated by Cox proportional hazards regression model.*

## Discussion

This retrospective cohort study found that appropriate PMB therapy resulted in a significantly lower 28-day mortality rate than appropriate TGC therapy or PMB/TGC combination therapy in patients with HAP caused by CRO. After the confounding factors were excluded, the survival advantage of PMB remained.

The findings of this study are consistent with some previous reports. Liang et al. (14) investigated the relationship between antibiotic treatment strategies and clinical outcomes in patients with CRAB-associated pneumonia. They found that TGC-based therapy was associated with higher ICU mortality than TGC-free therapy [adjusted odds ratio (aOR) 2.30, 95% CI 1.19–4.46]. In addition, the investigators found that colistin monotherapy was associated with lower ICU mortality when compared with TGC monotherapy (aOR 0.30, 95% CI 0.10–0.88). A similar trend was shown in treatment failure rates, i.e., TGC-based treatment was associated with a higher failure rate than non-TGC-based treatment (aOR 2.51, 95% CI 1.39–4.54). Kontopidou et al. investigated the relationship between antimicrobial treatment and patient outcomes. They reported that TGC-based therapies showed the highest failure and mortality rates (31). A multicenter prospective observational study found that when the MIC of TGC was greater than 2 mg/L, the colistin-combined TGC regimen was associated with increased 14-day mortality (32). In addition, TGC-based therapy was associated with lower microbial clearance and higher mortality (32). Another *in vitro* one-compartment dynamic model (IVM) study demonstrated the importance of the TGC dosing for the efficacy of PMB-TGC combination in the treatment of multidrug-resistant *A. baumannii*. Because the synergistic effect could only be achieved when PMB (1 mg/kg/12 h) was used in combination with a higher dose of TGC (200 mg/12 h instead of 100 mg/12 h) (33).

In fact, it is always a focus of debate among clinicians regarding combination therapy for patients with CRO infections. Other authors analyzed 22 studies comparing polymyxin monotherapy with polymyxin-based combination therapies in adult patients with infections caused by carbapenem-resistant Gram-negative bacteria (15). They found that polymyxin monotherapy was associated with significantly higher mortality than the therapies combined with aminoglycoside, fosfomycin, or TGC (unadjusted OR 1.57, 95% CI 1.06–2.32). However, the authors believed that the observed significant association between polymyxin monotherapy and mortality could not be taken as proof to support the advantages of combination therapy due to very low-quality evidence, such as selection bias and not controlled. The RCTs had shown that rifampicin/colistin (11), colistin/meropenem (12), or fosfomycin/colistin (34) had no effect on the mortality of CRO infections. As a number of patients were complicated with sepsis, we used the time to treatment as one of the criteria to determine the appropriateness of antimicrobial therapy (26). We also strictly limited the duration and timing of antimicrobial treatment, especially the strict definition of combination therapy. Therefore, our findings also supported to some extent the conclusion that combination therapy when compared to monotherapy may not necessarily confer a benefit as it is reliant on multiple factors, such as the choice of the partner antibiotics. We found that patients who received PMB/TGC combination therapy did not achieve mortality benefit, which suggests that TGC might not be suitable for combination with PMB.

Tigecycline has limitations in the treatment of HAP. This may be an important cause of increased mortality of TGC-based regimens. First of all, TGC is a bacteriostatic rather than bactericidal agent (35). Secondly, the concentration of TGC in plasma is low. The steady volume of distribution of TGC is about 7–10 L/kg indicating extensive distribution into body tissues (35). A standard TGC regimen (i.e., 100 mg loading dose followed by 50 mg maintenance dose) results in a maximum plasma concentration of only 0.6 μg/ml at a steady state (36). The low plasma concentration and lack of bactericidal effect may lead to a poor microbial response to TGC treatment. Shen et al. (37) found that the TGC treatment group achieved a lower microbiological success rate than the control group (OR = 0.94, 95% CI = 0.77–1.16, *p* = 0.56) and lower microbial clearance rate for *E. coli* and *K. pneumoniae*. Gardiner et al. (38) also found that the TGC treatment group had a significantly higher prevalence of bacteremia than control group 24 h after the start of treatment (*p* = 0.02). High-dose TGC might be able to make up the pharmacokinetic-pharmacodynamic disadvantages to some extent. A systematic review analyzed 25 studies regarding the effectiveness and/or safety of TGC-based regimens in the treatment of CRE infections. A subgroup meta-analysis of this review found that intensive care with high-dose TGC was associated with a much lower mortality rate than standard dose of TGC (OR 12.48; 95% CI 2.06–75.43; *p* = 0.006) (39).

As early as 2010, the United States FDA warned that TGC may cause increased mortality (40), especially when TGC is used to treat HAP (off-label use). Prasad et al. showed that TGC was associated with increased mortality [risk difference (RD) 0.7%; 95% CI 0.1–1.2%; *p* = 0.01] and increased non-cure rate (RD 2.9%; 95% CI 0.6–5.2%; *p* = 0.01) (41). This conclusion is independent of infection type, study design, and sample size. This may be due to the fact that TGC is bacteriostatic rather than bactericidal for the pathogens at lower plasma concentrations (41). Paul et al. analyzed 13 TGC clinical trials and found that TGC was associated with higher all-cause mortality rates than in the control group in 12 of the trials, especially for the ventilator-associated pneumonia (VAP) patients with baseline bacteremia (42). In patients with complicated skin/skin-structure infection, complicated intra-abdominal infection, or community-acquired bacterial pneumonia, which are approved indications, TGC is generally safer and well tolerated, with a cure rate similar to standard therapies (38).

The synergistic effect between PMB and TGC is not prominent *in vitro*, which is another important reason for the higher mortality of PMB/TGC combination. Wentao et al. found that PMB/TGC combination was synergistic in only 9.5% of the cases (95% CI 6.0–14.5%), but polymyxins/rifampicin combination was synergistic in 60.3% of the cases (95% CI 34.4–81.5%) (43). Chaoe et al. also found that the synergistic effect between colistin and TGC was about 20%, and no synergistic effect on the *E. coli* producing bla_*NDM*–1_ and *Serratia marcescens* (44).

In this study, we concluded that patients in the PMB/TGC combination group did not show a survival advantage as compared to patients in monotherapy groups (PMB group and TGC group). This might be related to the following reasons. In this study, patients in monotherapy groups, which served as the control group, might be concomitantly receiving other antibiotics (for example, carbapenems). Although these antibiotics were defined as inappropriate in this study, they might still have an impact on treatment efficacy, even if it might be mild, resulting in a lower mortality rate in PMB group when compared with that in the PMB/TGC combination group. In addition, the susceptibility test results of some target strains in this study were missing and the difference in the MIC value of the pathogenic strains between the combination group and the control groups was not clear. This could also be a reason why combination therapy did not appear to have a benefit. Finally, the antibacterial activity of TGC was insufficient, low local drug concentration in infection sites, possible direct drug toxicity, and other mechanisms might also contribute to higher mortality with the combination regimen than with the monotherapy (41).

Polymyxin B-based therapies were associated with AKI in this study. This is consistent with the conclusion of a recent study on polymyxin nephrotoxicity (45), which demonstrated that the incidence of nephrotoxicity was about 39.1% in the patients treated with intravenous polymyxin. Higher incidence of AKI may be related to the older age of patients, pre-existing AKI, and combined use of vasopressor. In addition, the patients included in our study were critically ill and usually received multiple drugs, which may be potentially toxic to kidneys.

There are some limitations to this study. First of all, this study is a retrospective observational study with inherent shortcomings, e.g., the investigators have known the specific treatment measures received by the patients before data analysis (classification bias) or investigators may tend to select cases that have an expected effect on the results of the study (selection bias). This study concludes that an appropriate PMB/TGC combination is associated with increased mortality as compared to appropriate PMB alone. However, more data and further studies are required to clarify whether this conclusion is widely applied as the limitation of the difference in the severity of disease among groups in this study. Treatment outcomes are affected not only by antimicrobial therapies but also by host factors (e.g., underlying diseases, disease severity) and pathogen factors (e.g., MIC of the isolates, mixed infection, and presence of heteroresistance). The population in this study was mainly critically ill patients with multiple bacterial infections, and susceptibility testing results were not collected completely. These limitations made it impossible to perform multivariate analysis on specific bacterial species and prevented the analysis to examine MIC effects on mortality. All the *P. aeruginosa* strains were isolated from mixed infection. Heteroresistance can also lead to the failure of combination therapy (46). Unfortunately, it was impossible to obtain the pathogenic isolates to analyze heteroresistance due to the retrospective nature of this study.

In summary, our findings suggest that the appropriate PMB/TGC combination was not superior to appropriate PMB therapy in the treatment of HAP caused by CRE/CRAB in terms of 28-day mortality. An RCT is urgently needed to confirm this preliminary finding. Renal function should be monitored closely during PMB therapy.

## Data Availability Statement

The original contributions presented in the study are included in the article/Supplementary Material, further inquiries can be directed to the corresponding author.

## Ethics Statement

The studies involving human participants were reviewed and approved by the Ethics Committee of China-Japan Friendship Hospital. Written informed consent for participation was not required for this study in accordance with the national legislation and the institutional requirements.

## Author Contributions

BC and KC conceived and designed the study. JZ, XY, BW, WS, MH, ZC, HC, YS, PC, XC, XGa, WM, LX, YW, XGu, and XZ collected the data. KC and HW analyzed and interpreted the data. KC drafted the manuscript. BC revised the manuscript. All authors agreed to be accountable for all aspects of the work in ensuring that questions related to the accuracy or integrity of any part of the work are appropriately investigated and resolved.

## Conflict of Interest

The authors declare that the research was conducted in the absence of any commercial or financial relationships that could be construed as a potential conflict of interest.

## Publisher’s Note

All claims expressed in this article are solely those of the authors and do not necessarily represent those of their affiliated organizations, or those of the publisher, the editors and the reviewers. Any product that may be evaluated in this article, or claim that may be made by its manufacturer, is not guaranteed or endorsed by the publisher.
