# Supplementary material for: Polymyxin B/Tigecycline Combination vs. Polymyxin B or Tigecycline Alone for the Treatment of Hospital-Acquired Pneumonia Caused by Carbapenem-Resistant Enterobacteriaceae or Carbapenem-Resistant Acinetobacter baumannii
Source: Front Med (Lausanne). 2022 Jun 10;9:772372. doi: 10.3389/fmed.2022.772372 (PMC9226555; doi:10.3389/fmed.2022.772372)
Supplement: Supplementary file 1 [file Data_Sheet_1.docx]

Supplementary Material

**Supplementary Figures and Tables**

**Table S1. Underlying diseases and concomitant infections of patients in terms of polymyxin B and/or tigecycline treatment**

| **Underlying disease and concomitant infection** | **Polymyxin B**  **(N = 99)** | **Tigecycline**  **(N = 173)** | **Polymyxin B + Tigecycline**  **(N = 92)** | ***P* value** |
| --- | --- | --- | --- | --- |
| Underlying disease and condition | | | | |
| Hospitalization within 30 days before this admission | 61 (61.6) | 86 (49.7) | 65 (70.7) | 0.003 |
| Diabetes mellitus | 15 (15.2) | 41 (23.7) | 23 (25.0) | 0.17 |
| Malignant tumor | 6 (6.1) | 7 (4) | 16 (17.4) | 0.0005 |
| Lung transplantation | 14 (14.1) | 9 (5.2) | 1 (1.1) | 0.0008 |
| Other organ transplants | 0 (0) | 3 (1.7) | 5 (5.4) | 0.03 |
| Long-term glucocorticoid therapy in the past month^*^ | 4 (4.0) | 8 (4.6) | 4 (4.3) | 1.00 |
| Chemotherapy within the last 3 months | 3 (3.0) | 2 (1.2) | 4 (4.3) | 0.25 |
| Concomitant infection other than HAP | | | | |
| Ventilator-associated pneumonia | 53 (53.5) | 81 (46.8) | 46 (50.0) | 0.56 |
| Bloodstream infection | 15 (15.2) | 22 (12.7) | 26 (28.3) | 0.005 |
| Urinary tract infection | 16 (16.2) | 7 (4.0) | 6 (6.5) | 0.002 |
| Intra-abdominal infection | 6 (6.1) | 5 (2.9) | 6 (6.5) | 0.25 |
| Skin and soft tissue infection | 1 (1) | 3 (1.7) | 4 (4.3) | 0.33 |
| Surgical site infection | 2 (2) | 8 (4.6) | 3 (3.3) | 0.58 |
| Other infection | 1 (1.0) | 0 (0.0) | 2 (2.2) | 0.09 |

Data are presented as mean ± standard deviation or N (%).

*Received prednisone > 5 mg/d (or equivalent dose) or other immunosuppressive drugs > 14 days in the past month.

**Table S2. Univariate Cox regression analysis of 28-day mortality**

| Variable | Survivors  (N = 223) | Deaths  (N = 141) | HR (95% CI) | P value |
| --- | --- | --- | --- | --- |
| Treatment group |  |  |  |  |
| Polymyxin B | 71 (71.7) | 28 (28.3) | 0.50 (0.31-0.81) | .004 |
| Tigecycline | 105 (60.6) | 68 (39.3) | 0.77 (0.53-1.12) | .169 |
| Polymyxin B/Tigecycline | 47 (51.1) | 45 (48.9) | 1.0 |  |
| Demographics | | | | |
| Sex |  |  |  |  |
| Male | 163 (59.5) | 111 (40.5) | 1.0 |  |
| Female | 60 (66.7) | 30 (33.3) | 0.79 (0.53-1.19) | .259 |
| Age, years | 61.00 ± 17.00 | 64.00 ± 24.00 | 1.01 (1.00-1.02) | .319 |
| Body mass index (kg/m^2^) | 22.87 ± 4.69 | 22.04 ± 4.77 | 0.99 (0.94-1.04) | .574 |
| Underlying disease | | | | |
| Chronic respiratory disease |  |  |  |  |
| No | 120 (61.9) | 74 (38.1) | 1.0 |  |
| Yes | 103 (60.6) | 67 (39.4) | 1.08 (0.77-1.50) | .670 |
| Chronic cardiovascular disease | | | | |
| No | 118 (69.4) | 52 (30.6) | 1.0 |  |
| Yes | 105 (54.1) | 89 (45.9) | 1.69 (1.2-2.39) | .003 |
| Chronic kidney disease |  |  |  |  |
| No | 195 (61.9) | 120 (38.1) | 1.0 |  |
| Yes | 28 (57.1) | 21 (42.9) | 1.14 (0.71-1.81) | .591 |
| Malignant tumor |  |  |  |  |
| No | 204 (60.9) | 131 (39.1) | 1.0 |  |
| Yes | 19 (65.5) | 10 (34.5) | 0.89 (0.47-1.69) | 0.71 |
| Bloodstream infection |  |  |  |  |
| No | 190 (63.1) | 111 (36.9) | 1.0 |  |
| Yes | 33 (52.4) | 30 (47.6) | 1.35 (0.90-2.02) | 0.14 |
| Severity of illness at diagnosis of HAP due to CROs | | | | |
| SOFA score | 5.00 ± 5.00 | 7.00 ± 5.00 | 1.12 (1.07-1.16) | <.001 |
| APACHE II score | 15.00 ± 10.00 | 19.00 ± 9.00 | 1.07 (1.05-1.10) | <.001 |
| Vasopressor use | 53 (23.8) | 51 (36.2) | 1.76 (1.25-2.49) | .001 |
| WBC count (×10^12^/L) | 10.64 ± 7.04 | 11.05 ± 8.21 | 1.02 (0.99-1.04) | .182 |
| Platelet count (×10^9^/L) | 158.50 ± 147.00 | 124.00 ± 140.00 | 0.998 (0.996-0.999) | .010 |
| Creatinine (μmol/L) | 61.35 ± 55.05 | 83.30 ± 75.00 | 1.00 (1.00-1.00) | .064 |
| Bilirubin (μmol/L) | 15.30 ± 16.56 | 14.50 ± 14.43 | 1.00 (1.00-1.00) | .979 |
| PaO_2_/FiO_2_ ratio | 224.07 ± 153.55 | 179.75 ± 153.60 | 0.998 (0.996-0.999) | .009 |
| Sepsis |  |  |  |  |
| Yes | 89 (60.5) | 58 (39.5) | 1.51 (0.99-2.30) | .054 |
| Septic shock | 44 (47.8) | 48 (52.2) | 2.48 (1.60-3.83) | <.001 |
| No | 90 (72.0) | 35 (28.0) | 1.0 |  |
| Pathogens | | | | |
| Carbapenem-resistant *K. pneumoniae* | | | | |
| No | 83 (56.5) | 64 (43.5) | 1.0 |  |
| Yes | 140 (64.5) | 77 (35.5) | 0.74 (0.53-1.03) | .071 |
| Carbapenem-resistant *E. coli* |  |  |  |  |
| No | 214 (61.5) | 134 (38.5) | 1.0 |  |
| Yes | 9 (56.3) | 7 (43.8) | 1.21 (0.57-2.59) | .618 |
| Other CRE |  |  |  |  |
| No | 190 (59.6) | 129 (40.4) | 1.0 |  |
| Yes | 33 (73.3) | 12 (26.7) | 0.63 (0.35-1.14) | .127 |
| Carbapenem-resistant *A. baumannii* | | | | |
| No | 76 (65.0) | 41 (35.0) | 1.0 |  |
| Yes | 147 (59.5) | 100 (40.5) | 1.12 (0.78-1.61) | .537 |
| Carbapenems-resistant *P. aeruginosa* | | | | |
| No | 151 (60.9) | 97 (39.1) | 1.0 |  |
| Yes | 72 (62.1) | 44 (37.9) | 0.95 (0.67-1.36) | .797 |
| Number of pathogens |  |  |  |  |
| 1 | 83 (63.8) | 47 (36.2) | 1.0 |  |
| ≥ 2 | 140 (59.8) | 94 (40.2) | 1.12 (0.79-1.58) | .543 |
| Outcomes |  |  |  |  |
| AKI before infection |  |  |  |  |
| No | 180 (80.7) | 102 (72.3) | 1.0 |  |
| Yes | 43 (19.3) | 39 (27.7) | 1.52 (1.05-2.19) | .027 |
| AKI after infection |  |  |  |  |
| No | 142 (63.7) | 63 (44.7) | 1.0 |  |
| Yes | 81 (36.3) | 78 (55.3) | 1.81 (1.30-2.52) | .0005 |
| Clinical success |  |  |  |  |
| No | 100 (45.2) | 137 (97.2) | 1.0 |  |
| Yes | 121 (54.8) | 4 (2.8) | 0.04 (0.01-0.11) | <.0001 |
| Microbiological clearance |  |  |  |  |
| No | 126 (56.5) | 78 (55.3) | 1.0 |  |
| Yes | 97 (43.5) | 63 (44.7) | 1.07 (0.77-1.49) | 0.69 |

Abbreviations: HR, hazard ratio; CI, confidence interval; CRO, carbapenem-resistant organism; HAP, hospital-acquired pneumonia; SOFA, sequential organ failure assessment. WBC, white blood cell; APACHE, acute physiology and chronic health evaluation; PaO_2_, arterial oxygen partial pressure; FiO_2_, fractional inspired oxygen.

Data are presented as mean ± standard deviation or N (%).

Variables with missing values are presented as the number of patients evaluated.

**Table S3. The existing susceptibility testing results for the target strains.**

|  |  |  | Polymyxin B | Tigecycline | Polymyxin B/Tigecycline | P value |
| --- | --- | --- | --- | --- | --- | --- |
| Carbapenem-resistant A. baumannii | Polymyxin B | S | 0 (0.0) | 0 (0.0) | 0 (0.0) | --- |
|  |  | I | 22 (100) | 27 (100) | 23 (100) |  |
|  |  | R | 0 (0.0) | 0 (0.0) | 0 (0.0) |  |
|  | Tigecycline | S | 10 (35.7) | 40 (72.7) | 25 (67.6) | 0.0053 |
|  |  | I | 9 (32.1) | 12 (21.8) | 8 (21.6) |  |
|  |  | R | 9 (32.1) | 3 (5.5) | 4 (10.8) |  |
| Carbapenem-resistant P. aeruginosa | Polymyxin B | S | 0 (0.0) | 0 (0.0) | 0 (0.0) |  |
|  |  | I | 12 (100) | 2 (100) | 5 (100) | --- |
|  |  | R |  |  |  |  |
| Carbapenem-resistant K. pneumoniae | Polymyxin B | S | 0 (0.0) | 0 (0.0) | 0 (0.0) | 0.306 |
|  |  | I | 10 (100) | 13 (100) | 9 (90) |  |
|  |  | R | 0 (0) | 0 (0) | 1 (10) |  |
|  | Tigecycline | S | 11 (37.9) | 27 (69.2) | 20 (76.9) | 0.0092 |
|  |  | I | 11 (37.9) | 8 (20.5) | 1 (3.8) |  |
|  |  | R | 7 (24.1) | 4 (10.3) | 5 (19.2) |  |

Abbreviations: S, sensitive; I, intermediate; R, resistant.

Data are presented as N (%).

**Table S4. Patient enrollment in each participating hospital**

| **Hospital** | **Province** | **Number of patients** |
| --- | --- | --- |
| China-Japan Friendship Hospital | Beijing | 66 |
| Fujian Medical University Union Hospital | Fujian | 15 |
| The Second Xiangya Hospital of Central South University | Hunan | 18 |
| Jiangsu Provincial Hospital Affiliated to Nanjing Medical University of Medicine | Jiangsu | 32 |
| Wuxi People's Hospital | Jiangsu | 33 |
| Zhejiang Provincial People's Hospital | Zhejiang | 35 |
| The Second Affiliated Hospital of Zhejiang University School of Medicine | Zhejiang | 28 |
| Tongji Hospital affiliated to Tongji Medical College, Huazhong University of Science and Technology | Hubei | 41 |
| Union Hospital affiliated to Tongji Medical College, Huazhong University of Science and Technology | Hubei | 10 |
| Zhongnan Hospital of Wuhan University | Hubei | 25 |
| The First Affiliated Hospital of Zhengzhou University | Henan | 7 |
| The First Affiliated Hospital of Nanchang University | Jiangxi | 10 |
| Zhongshan Hospital of Fudan University | Shanghai | 21 |
| Ruijin Hospital Affiliated to Shanghai Jiao Tong University Medical School | Shanghai | 23 |
